# Effect of Halide Anions on Electrochemical CO_2_ Reduction in Non‐Aqueous Choline Solutions using Ag and Au Electrodes

**DOI:** 10.1002/open.202400166

**Published:** 2024-09-10

**Authors:** Hengameh Farahmandazad, Simone Asperti, Ruud Kortlever, Earl Goetheer, Wiebren de Jong

**Affiliations:** ^1^ Section of Large Scale Energy Storage Process & Energy Department Faculty of Mechanical Engineering Delft University of Technology Leeghwaterstraat 39 2628 CB Delft The Netherlands

**Keywords:** Electrochemical CO_2_ reduction, Gold electrode, Halide anion effect, Non-aqueous electrolyte, Silver electrode

## Abstract

In this study, the effect of halide anions on the selectivity of the CO_2_ reduction reaction to CO was investigated in choline‐based ethylene glycol solutions containing different halides (ChCl : EG, ChBr : EG, ChI : EG). The CO_2_RR was studied using silver (Ag) and gold (Au) electrodes in a compact H‐cell. Our findings reveal that chloride effectively suppresses the hydrogen evolution reaction and enhances the selectivity of carbon monoxide production on both Ag and Au electrodes, with relatively high selectivity values of 84 % and 62 %, respectively. Additionally, the effect of varying ethylene glycol content in the choline chloride‐containing electrolyte (ChCl : EG 1 : X, X=2, 3, 4) was investigated to improve the current density during CO_2_RR on the Ag electrode. We observed that a mole ratio of 1 : 4 exhibited the highest current density with a comparable faradaic efficiency toward CO. Notably, an evident surface reconstruction process took place on the Ag surface in the presence of Cl^−^ ions, whereas on Au, this phenomenon was less pronounced. Overall, this study provides new insights into anion‐induced surface restructuring of Ag and Au electrodes during CO_2_RR, and its consequences on the reduction performance on such surfaces in non‐aqueous electrolytes.

## Graphical abstract

The choice of electrolyte and electrode material is of importance in determining the product selectivity. The presence of specific anions also significantly influences CO_2_RR product selectivity. In choline‐halide ethylene glycol electrolytes, the Ag electrode outperforms the Au electrode in CO selectivity. Among the halides, Cl^−^ notably enhances CO partial current density and leads to an evident surface reconstruction of the Ag surface.

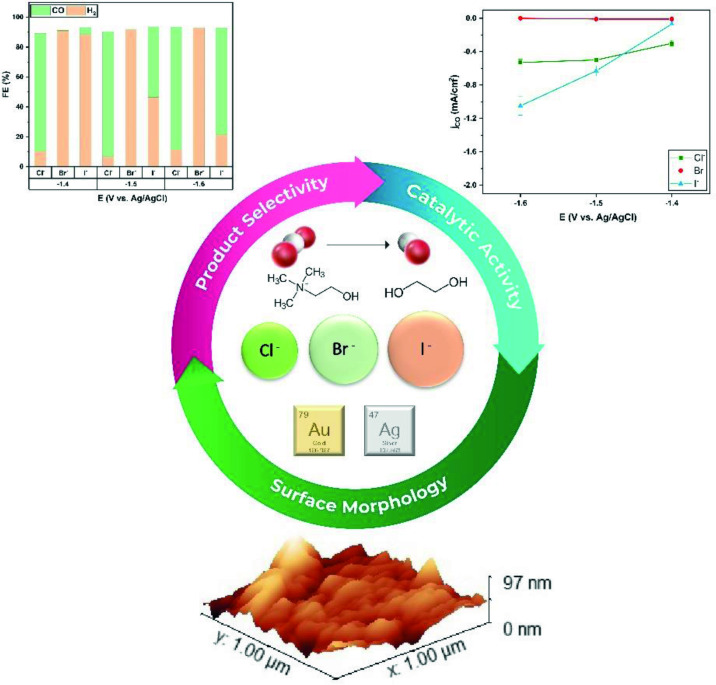

## 1 Introduction

In recent times, carbon capture and utilization (CCU) technologies have gained interest as innovative approaches to mitigate CO_2_ emissions, offering both environmental and economic benefits.[^1^, ^2^] Among these, electrochemical CO_2_ reduction reaction (CO_2_RR) stands out as a promising method of CCU approaches to convert CO_2_ into valuable chemicals and fuels, driven by renewable energy sources like solar or wind power. This approach has attracted significant attention in the past few decades leading to a significant number of studies focused on developing the CO_2_RR toward industrial applications.[^3^, ^4^, ^5^] However, challenges such as low product selectivity, catalyst instability, and in some cases low activity are currently hampering its large‐scale application.^[6]^ To address these issues, significant research efforts have been dedicated to improving electrocatalysts,^[7]^ electrolytes,[^8^, ^9^] and electrolyzers,^[10]^ with a focus on reducing overpotentials and enhancing selectivity toward commercially important products like carbon monoxide, ethylene, methane, methanol, formic acid, higher molecular weight carboxylic acids, and aldehydes.[^11^, ^12^]

Particularly, carbon monoxide (CO) is considered a promising product as it can be used as intermediate in downstream chemical transformations. CO can also be used in combination with hydrogen (forming syngas) for industrial applications, with an annual global production of approximately 150 Mtonnes, reported in 2018.^[13]^

The choice of electrode material plays a critical role in determining product selectivity in CO_2_RR. Silver (Ag) and gold (Au) electrodes are known to exhibit high selectivities toward CO, attributed to their weak CO binding, hindering further reduction of CO, and facilitating easy desorption of CO* from the surface.[^14^, ^15^, ^16^]

Furthermore, the interaction between electrodes and electrolytes significantly influences electrocatalytic performance.[^8^, ^17^, ^18^, ^19^, ^20^] Electrolytes can influence the electrocatalytic performance by changing the surface morphology as a result of electrode‐electrolyte interactions, impacting product distribution.[^18^, ^21^] Moreover, the electrolyte properties including the cation and anion species used, their overall concentration, CO_2_ solubility, electrical conductivity, viscosity, and electrolyte pH influence the local reaction conditions at the electrocatalytic surface and thus the product distribution and performance of the electrocatalyst.[^15^, ^19^, ^20^, ^22^, ^23^]

Electrolytes for CO_2_RR can be roughly categorized as aqueous and non‐aqueous solvents. Aqueous electrolytes allow for hydrocarbon production; however, often the abundant presence of water and protons promotes the hydrogen evolution reaction (HER). Therefore, the faradaic efficiency (FE) values toward CO_2_ reduction products remains often limited.[^9^, ^24^] Moreover, the solubility of CO_2_ in aqueous electrolytes at atmospheric pressure is low (≈34 mM), which constrains the amount of CO_2_ dissolved in the electrolyte and, consequently, limits the CO_2_RR.[^9^, ^25^] In contrast, non‐aqueous electrolytes offer higher CO_2_ solubility and lower proton and water concentrations, suppressing HER and enhancing CO_2_RR efficiency.[^8^, ^9^, ^26^, ^27^] Nevertheless, the presence of water is, most of the times, unavoidable, since water can crossover from the aqueous anolyte – when used – to the catholyte. While most non‐aqueous electrolytes can effectively suppress the HER, a small amount of water, serving as a proton donor, can enhance current densities for CO_2_RR.^[28]^ Furthermore, water can be generated during the CO_2_ reduction reaction, following the widely accepted mechanistic pathway for CO formation. Over either Ag or Au electrodes, the initial proton and electron transfer to the dissolved CO_2_ forms an adsorbed species *COOH (Eqs. (1)–(2)). This intermediate species eventually results in CO formation, once a proton and electron transfers to the hydroxyl group in *COOH form and produces water.[^14^, ^25^, ^29^]
(Eq. 1)

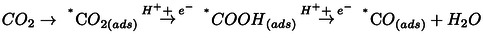



(Eq. 2)

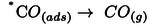




Some non‐aqueous electrolytes face challenges due to their relatively low electrical conductivity and high viscosity, which can be overcome using additives such as ionic liquids (IL), deep eutectic solvents (DES), organic salts, and other ionic species.^[24]^ These additives affect the electrochemical process by enhancing the CO_2_ concentration at the electrode surface, altering the local electric field, adjusting intermediates’ adsorption energy, and causing changes in surface morphology.^[24]^ The presence of ionic species plays an impacting role on the electrochemical process, and their contribution depends on (I) the size of ionic species; (II) the specific adsorption of ions on the metallic electrodes; (III) their hydration strength; and (IV) the intrinsic physical and chemical properties of ions in the electrolyte.[^21^, ^24^] Interestingly, their effect is visible on the electrode surface and changes the catalyst morphology during CO_2_RR.^[24]^

For instance, studies by Rosen et al. showed that ionic liquids can significantly lower the onset potentials of CO_2_RR, suppressing the HER on silver electrodes.^[30]^ Gao et al. observed how the presence of halide anions can lower overpotential and heighten the selectivity of CO_2_RR, attributing this enhancement to their specific adsorption on the surface of copper electrodes.^[31]^ Similarly, Huang et al. observed increased faradaic efficiencies and partial current densities for ethylene and ethanol on copper electrodes in the presence of halides, particularly iodide in aqueous solutions.^[32]^ Moreover, research by Zhou et al. highlighted the effect of chloride‐containing ILs in reducing CO_2_ to CO in aqueous electrolytes, suppressing HER driven by water‐anion and water‐cation interactions.^[33]^

While deep eutectic solvents share similarities with IL‐based systems in facilitating CO_2_RR, they offer distinct advantages such as abundance, biodegradability, affordability, ease of preparation, and environmental friendliness.^[34]^ These attributes make deep eutectic solvents attractive for various applications including CO_2_ capture[^34^, ^35^] and serving as electrolytes[^36^, ^37^, ^38^] in CO_2_RR. Notably, the use of such solvents can limit water presence, thereby suppressing HER and enhancing CO_2_RR efficiency.^[39]^

Choline chloride (ChCl) and ethylene glycol (EG) can form a deep eutectic solvent and offer distinctive advantages for CO_2_ reduction reactions due to their properties and roles in catalysis.^[37]^ Previous studies have highlighted the co‐catalytic potential of choline chloride in non‐aqueous electrolytes, showing lowered onset potentials for reduction current and increased selectivity toward CO.^[37]^ By altering the electrochemical environment, choline chloride enhances catalytic activity and promotes selectivity toward desired CO_2_ reduction products.^[18]^ Ethylene glycol, a commonly used solvent in industry, can function as a co‐catalyst or co‐solvent in CO_2_ reduction reactions, facilitating the solubilization of CO_2_ and enhancing its availability for the catalytic process. Additionally, ethylene glycol is relatively non‐toxic and widely available on an industrial scale further enhancing its application.[^39^, ^40^, ^41^] Therefore, their utilization holds promise for the development of more efficient and sustainable processes aimed at converting CO_2_ into valuable chemicals and fuels.

However, despite extensive research[^29^, ^32^, ^42^, ^43^, ^44^, ^45^] on the influence of specific anions on CO_2_RR product selectivity, the effect of halide anions in organic solvents on silver and gold electrodes remains largely unexplored. Therefore, to elucidate this effect, this study aims to investigate the CO_2_RR using Choline‐X (X=Cl^−^, Br^−^, I^−^) solutions in ethylene glycol as electrolytes, coupled with silver and gold electrodes in a compact H‐cell setup. Employing cyclic voltammetry (CV) and chronoamperometry (CA), the behavior of electrocatalytic reduction of CO_2_ to CO on both electrodes in selected solvents was examined, with further analysis conducted through scanning electron microscopy (SEM), energy‐dispersive X‐ray spectroscopy (EDS), atomic force microscopy (AFM), and inductively coupled plasma optical emission spectroscopy (ICP‐OES). Herein, we clearly demonstrate that Cl^−^ stands out as anion for the surface reconstruction of a flat silver surface, resulting also in higher FE's and partial current densities for CO production. Although much research is needed in the field of non‐aqueous solvents, we believe that the findings reported in this work will be helpful for the improvement of the CO_2_RR and, in turn, speed up the research in the field of CCU technologies.

## 2 Materials and Methods

### 2.1 Materials

Three different choline‐based salts – choline chloride (≥98 %, Sigma–Aldrich), choline bromide (>98.0 %, TCI), and choline iodide (>98.0 %, TCI) – were procured from commercial suppliers. These salts were used as hydrogen bond acceptors (HBA) and dissolved in ethylene glycol (≥99.5 %, for analysis, EMSURE), serving as a hydrogen bond donor (HBD). The prepared deep eutectic solvents were utilized as catholytes, while a 0.5 M aqueous solution of sulfuric acid (95–98 %, ACS reagent, Sigma–Aldrich) was employed as an anolyte.

For the electrodes, a silver (Ag) foil (1.0×25×25 mm, MaTeck, 99.9 % metal basis) and a gold (Au) foil (1.0×25×25 mm, MaTeck 99.99 % metal basis) served as working electrodes, while a platinum (Pt) foil (0.1×25×25 mm, MaTeck, 99.99 % metal basis) was utilized as the counter electrode. The Ag electrode underwent sanding with silicon carbide grinding papers of varying roughness (#80, 180, 320, 800,1200, 2000, SiC paper, Struers), followed by polishing to a mirror‐like finish using diamond suspensions and alumina pastes. Subsequently, the electrodes were sonicated in an ultrasonic bath (M2800H, Bransonic) to eliminate any traces of the polishing pastes and rinsed with ultrapure water (resistivity 18.2 MΩ ⋅ cm at 25 °C, Q‐POD1, Merck Milli‐Q) and dried with air. Organic impurities deposited on the surfaces of the Au and Pt electrodes were removed through flame annealing before each experiment.

A leak‐free Ag/AgCl reference electrode (1 mm OD, LF‐1, Innovative Instruments), stored in a 3 M KCl solution, served as reference electrode. A cation exchange membrane Nafion™ 117 (thickness 0.007 in, perfluorinated, Sigma–Aldrich), stored in ultrapure water, was utilized to separate the anolyte and catholyte cell compartments.

High‐purity nitrogen and carbon dioxide gases (5 and 4.5 purity, Linde Gas Benelux B.V.) were supplied for the experiments. All procedures were conducted at room temperature and pressure, and each experiment was performed in duplicate, with reported results being averaged.

### 2.2 Electrolyte Preparation

**Figure 1:**
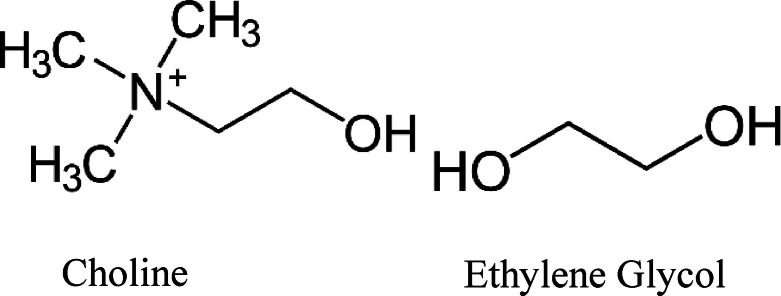
The structures of choline and ethylene glycol.

A series of choline‐based solvents with different anion structures (as depicted in Figure 1) were synthesized to serve as catholytes. The preparation of Choline Chloride : Ethylene Glycol (ChCl : EG) electrolytes followed the methodology outlined by Abbott et al.^[46]^ To ensure minimal water content and achieve a non‐aqueous solution, choline chloride underwent drying in a laboratory oven (E 28, Binder) at 110 °C for 2 hours.^[47]^ Subsequently, ChCl : EG solutions at mole ratios of (1 : 2), (1 : 3), and (1 : 4) were prepared by mixing dried choline chloride with ethylene glycol. This mixture was gently stirred and heated to 80 °C for 2 hours until a clear, colorless, and homogeneous liquid was obtained. After cooling to room temperature, the solution was stored under vacuum conditions to prevent water contamination between experiments.

Similar preparation methods were employed for Choline Bromide : Ethylene Glycol (ChBr : EG=1 : 4) and Choline Iodide : Ethylene Glycol (ChI : EG=1 : 4) electrolytes, with the temperature increased to 90 °C. For ChI : EG, the addition of 15 wt% water to the final solution was necessary to prevent crystal formation.

### 2.3 Characterization

The electrical conductivity of the electrolytes was measured using a pH/conductometer (914, Metrohm), while their viscosity was measured using a rotating viscometer (low‐shear 40, Contraves™). The water content was determined using a volumetric Karl–Fischer titrator (V10S, Mettler Toledo). All measurements were performed at room temperature and ambient pressure conditions. To analyze the elements present in the electrolyte, an inductively coupled plasma optical emission spectroscopy analyzer (ICP‐OES) (Spectro Arcos, Ametek) was utilized.

Before and after CO_2_RR, the surface of the silver and gold working electrodes underwent characterization using scanning electron microscopy and energy‐dispersive X‐ray spectroscopy (SEM‐EDS, Jeol 6500 F) to monitor surface morphology changes and surface impurities, respectively. The SEM operated with an electron beam energy of 15 kV and at a working distance of 25 mm. Additionally, surface topography measurements were conducted using an atomic force microscope (AFM – Dimension Edge Scanning Probe Microscope, Bruker). Surface scanning was performed in Tapping mode using a high‐sensitivity silicon probe (RTESPA‐300, Bruker).

### 2.4 Electrolysis Cell Configuration, Operation, and Analysis

Cyclic voltammetry (CV) measurements were performed in a three‐electrode H‐cell, where electrolytes were saturated either with N_2_ or CO_2_, using a scan rate of 50 mV/s. Controlled potential electrolysis (CPE) experiments were conducted via chronoamperometry (CA) in a custom compact three‐electrode divided electrochemical cell, as previously described.^[48]^ This cell, divided by a cation exchange membrane (Nafion™ 117), facilitated proton transfer from the acidic anolyte to the catholyte while preventing the transport of liquid products from the working electrode to the counter electrode, where they could be oxidized. Each cathode and anode compartment contains 1.8 mL electrolytes and 1 cm^2^ electrode surface area. The parallel plate electrode geometry ensures uniform voltage distribution over the catalyst surface.^[48]^ A leak‐free Ag/AgCl reference electrode was inserted in the cathode compartment to monitor the working electrode potential. The cell components, fabricated from PEEK, underwent cleaning with a 20 % (v/v) aqueous nitric acid solution, followed by rinsing with Milli‐Q water and drying with air and nitrogen.

Electrochemical experiments were carried out using either a single‐channel potentiostat (BioLogic SP‐200) or a multichannel potentiostat (BioLogic VSP 300), controlled by BioLogic's EC‐Lab® software (version 11.30). Faradaic efficiencies for products obtained during CPE experiments were calculated as the average of two reproducible results with their standard error. Potentiostatic electrochemical impedance spectroscopy (PEIS) was employed to determine resistance (R_u_), with all potentials reported versus the Ag/AgCl reference electrode.

For electrochemical measurements, the anode chamber was filled with a 0.5 M aqueous solution of sulfuric acid, left open to the atmosphere. Meanwhile, the cathode chamber was filled with the specific electrolyte under study and continuously purged with CO_2_ at a flow rate of 8 mL.min^−1^ for one hour during CPE experiments. The CO_2_ flow rate was regulated by a mass flow controller (F‐201CV/F‐211CV, Bronkhorst®) integrated into a gas flow meter upstream of the cell. CO_2_ purge was started 20 minutes before the experiments to ensure electrolyte saturation.

Gaseous products produced in the cathode chamber were directed to an in‐line gas chromatograph (GC) (Compact GC4.0, Global™ Analyzer Solutions G.A.S) for analysis. The GC was equipped with two thermal conductivity detector (TCD) channels and one flame ionization detector (FID) channel. It was calibrated in a concentration range from 50 to 8000 ppm for gas products, including hydrogen (H_2_), carbon monoxide (CO), methane (CH_4_), ethylene (C_2_H_4_), and ethane (C_2_H_6_), using calibration standards of these gasses in CO_2_ (Linde Gas Benelux B.V.).

Liquid products in both chambers were accumulated during the reaction. After the experiments, samples were collected for further analysis using high‐performance liquid chromatography (HPLC) (Agilent 1260 Infinity, Agilent Technologies). HPLC analysis involved injecting 5 μl of the solution onto two Aminex HPX‐87 H columns (Biorad) placed in series. The columns, heated to 60 °C, utilized an eluent containing 1 mM H_2_SO_4_ in ultrapure water, with a refractive index detector (RID) employed for product detection. Calibration of the HPLC was performed with standard aqueous solutions in a range of 0.1 mM to 50 mM for liquid products, including oxalic acid, glyoxal, formate, acetic acid, ethylene glycol, acetaldehyde, methanol, ethanol, acetone, propionaldehyde, 2‐propanol, 1‐propanol, choline chloride, and acetic acid.

## 3 Results and Discussion

### 3.1 Effect of Counter Ions

Understanding of how different halide anions in the choline halide and ethylene glycol electrolyte influence the performance of CO_2_ reduction reactions is crucial for advancing electrochemical processes in this system. We explored this by using various choline salts (ChX : EG, with X=Cl^−^, Br^−^, I^−^) and conducted cyclic voltammetry (CV) experiments to determine the potential range for CO_2_ reduction on Au and Ag electrodes.

**Figure 2:**
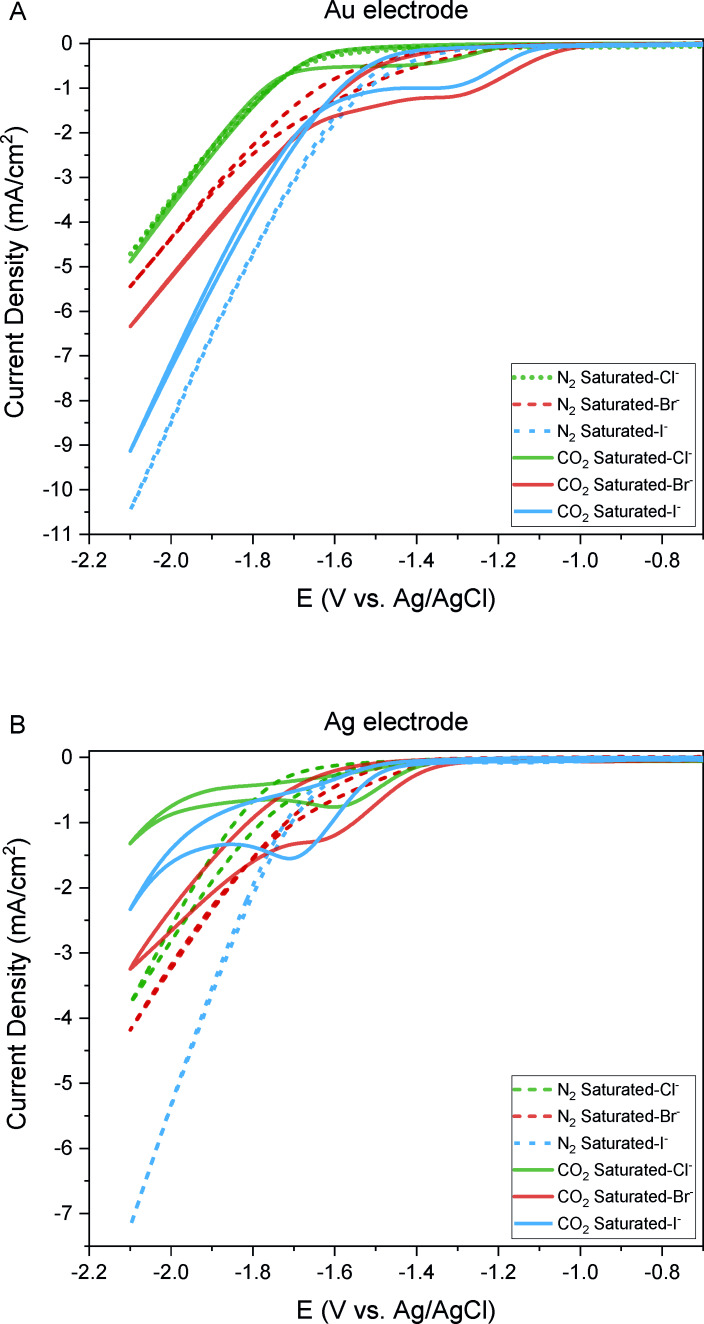
Cyclic voltammograms of A) Au foil electrode, and B) Ag foil electrode in ChX : EG 1 : 4 (X: Cl^−^, Br^−^, I^−^) electrolytes saturated with either N_2_ (dotted lines) or CO_2_ (solid lines).

We observed distinct behaviors in N_2_ and CO_2_ saturated electrolytes (Figure 2). In this figure, N_2_‐saturated solutions represented by dotted lines show a gradual decrease in current with increasingly negative potentials, indicative of the hydrogen evolution reaction. However, introducing CO_2_ which is represented by solid lines led to the appearance of a reduction peak, suggesting CO_2_ reduction reaction (Figure 2 and Table 1).

**Table 1:** Onset potentials of ChX : EG 1 : 4 (X: Cl^−^, Br^−^, I^−^) electrolytes saturated with N_2_ and CO_2_.

	E (V vs. Ag/AgCl)					
	Au			Ag		
	Cl^−^	Br^−^	I^−^	Cl^−^	Br^−^	I^−^
N_2_	−1.60	−1.35	−1.40	−1.70	−1.50	−1.40
CO_2_	−1.37	−1.31	−1.28	−1.57	−1.60	−1.70
Onset shift	0.23	0.04	0.12	0.13	−0.10	−0.30

**Figure 3:**
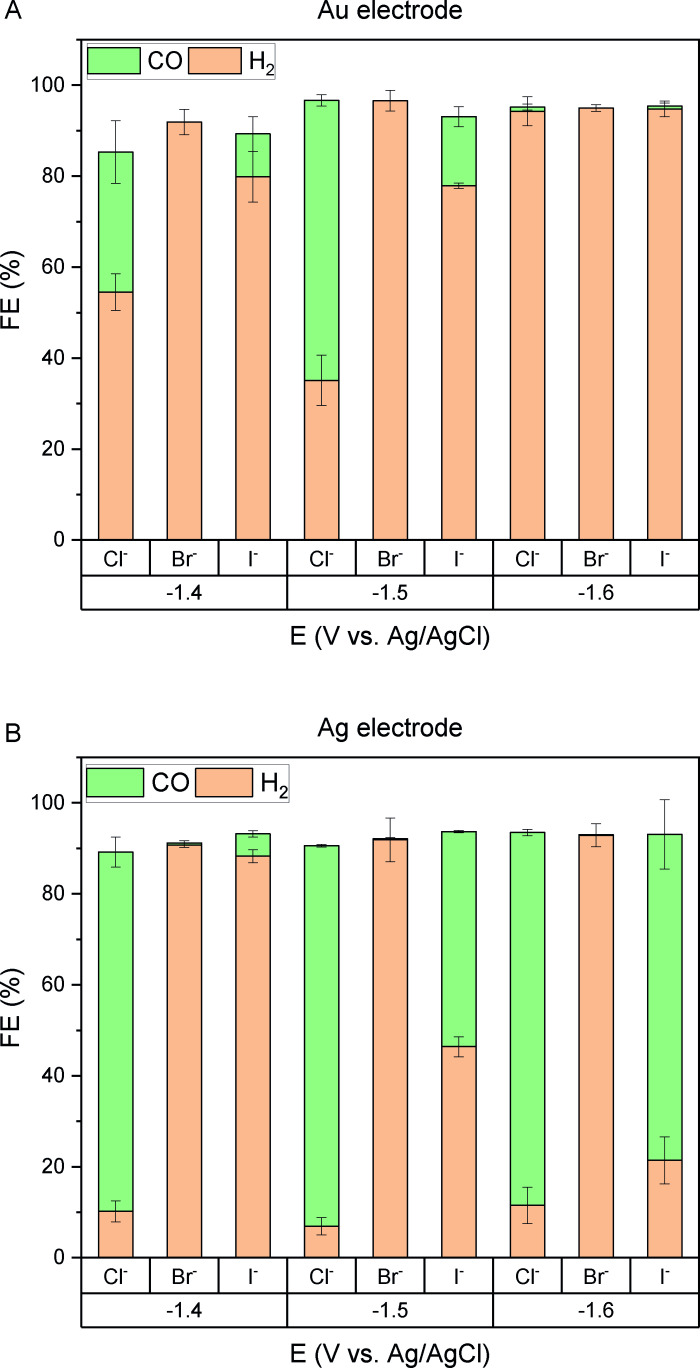
Faradaic efficiency for CO and H_2_ on A) Au electrode B) Ag electrode in ChX : EG 1 : 4 (X : Cl^−^, Br^−^, I^−^) catholytes after 1 hour of controlled potential electrolysis experiments.

To confirm this hypothesis, we further evaluated the CO_2_RR performances at specific reduction peak potentials (−1.4 V, −1.5 and −1.6 V vs. Ag/AgCl) for both silver and gold electrodes. Gas chromatography (GC) analysis confirmed the production of CO and H_2_ for the CO_2_ saturated electrolytes with varying selectivities, highlighting the influence of the electrolyte composition. (Figure 3 and Table S2 and S3). No other products such as formate or hydrocarbons were detected by either GC or HPLC (Figure S1). The generation of hydrogen stems from the availability of protons and water within the system. As previously mentioned, water can permeate into the catholyte through membrane crossover from the aqueous anolyte and is also produced during the CO_2_ reduction reaction (Eqs. (1) and (2)). The presence of water was quantified through Karl–Fischer titration conducted before and after the experiment (Table S1).

Interestingly, we found that the Ag electrode outperforms the Au electrode in terms of CO selectivity at all investigated potentials, regardless of the type of anion present in the electrolyte. However, it is important to note that the presence of different halide ions significantly impacted the CO selectivity and faradaic efficiency (FE_CO_). Specifically, Cl^−^ exhibited the highest FE_CO_ on both Ag and Au electrodes.

**Figure 4:**
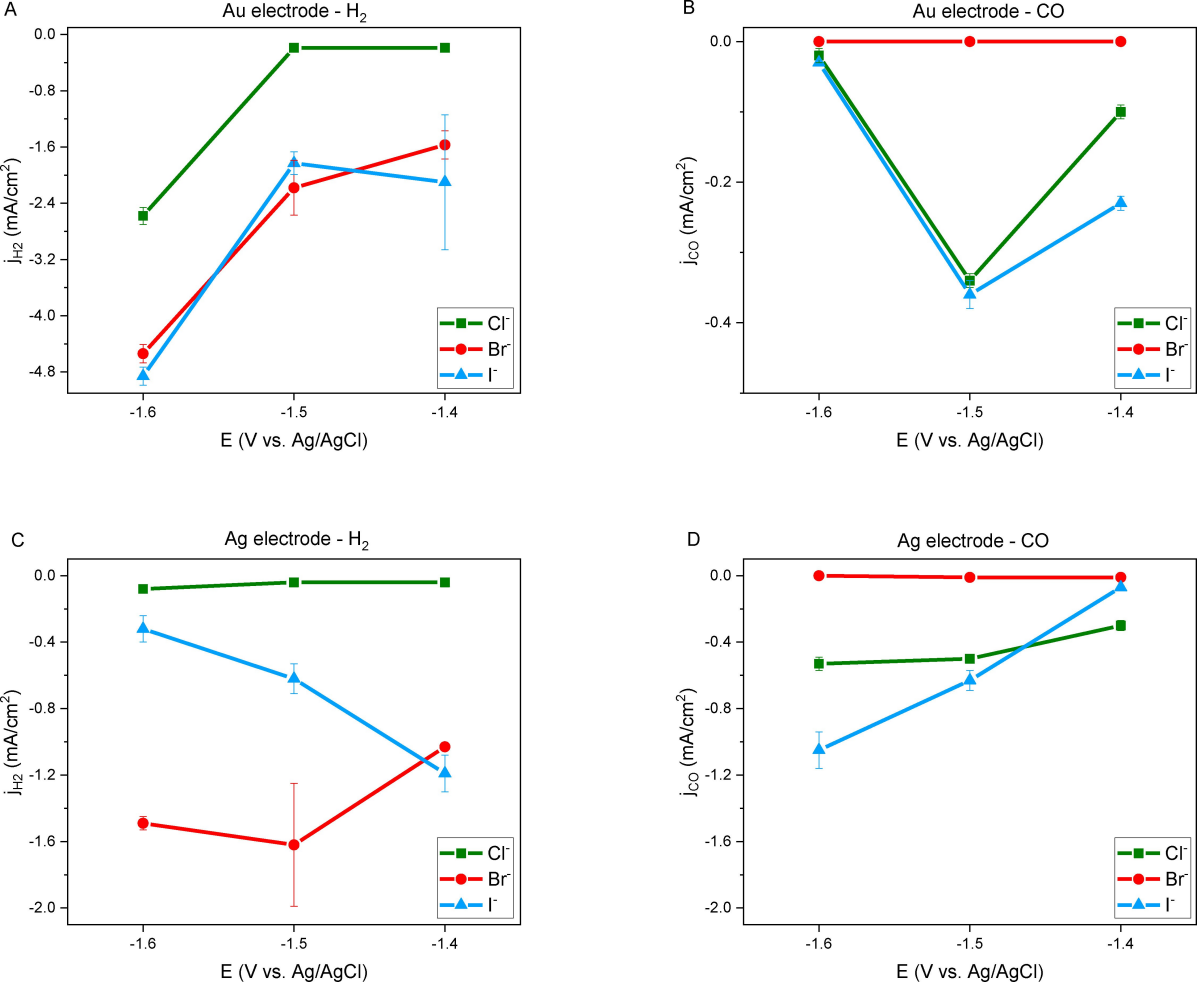
Partial current density vs. applied potential: A) Au electrode – H_2_ B) Au electrode – CO C) Ag electrode – H_2_ D) Ag electrode – CO in ChX : EG 1 : 4 (X : Cl^−^, Br^−^, I^−^) catholytes after 1 hour of controlled potential electrolysis experiments.

For the Ag electrode, a FE_CO_ exceeding 80 % was consistently observed at all three selected potentials in the presence of Cl^−^, while I^−^ demonstrated heightened CO selectivity with more negative potentials. Additionally, it is evident that the hydrogen evolution reaction occurred more prominently in the presence of I^−^. The observed trend for CO selectivity (Figure 3) and its partial current density (Figure 4) was found to be Cl^−^>I^−^>Br^−^, which aligns with findings by Garg et al. Despite their use of aqueous electrolytes, the trend remains similar.^[29]^ Furthermore, the obtained CO faradaic efficiency of 84 % in the presence of Cl^−^ is comparable to experimental observations by Vasilyev et al.^[37]^ and Garg et al.^[29]^ reporting 78 % and 94 % in ChCl : EG (1 : 2) and 0.1 M ChCl in water, respectively.

On the Au electrode, the highest CO selectivity within this potential range was achieved at −1.5 V vs. Ag/AgCl. When Cl^−^ was present in the solution, the FE_CO_ reached 62 %, whereas with I^−^, it was lower at the same potential (FE_CO_=15 %). Notably, on the Au electrode, the hydrogen evolution reaction is predominantly observed after 1 hour. Meanwhile, CO production on the Ag electrode steadily is increased over time and stabilized after 1 hour in the presence of Cl^−^ (Figure S3 and S5 and S6).

The observed trends are attributed to various factors, including the hydration strength of the ions, their adsorption on electrode surfaces, their effects on the double layer capacitance, and morphology changes driven by adsorbed reaction intermediate facilitation of atomic mobility (ARIAM) mechanism.[^18^, ^21^, ^23^, ^29^]

Smaller anions like Cl^−^ exhibit stronger hydration compared to larger counterparts such as Br^−^ and I^−^. This increased hydration restricts the presence of free water within the double layer, consequently suppressing the hydrogen evolution reaction.[^23^, ^29^] Conversely, larger anions tend to cover the catalyst electrode surface, occupying active sites and limiting the availability for CO_2_ reduction. This elucidates the remarkable enhancement of CO_2_RR and suppression of HER observed in the presence of Cl^−^.

For larger anions like I^−^, this observation (Figure 3 and 4) is attributed to a higher surface coverage of I^−^, resulting in a relatively lower CO partial current density. Additionally, the results of Karl‐Fischer titration experiments suggest that the enhanced HER activity with I^−^ may also stem from the additional and unavoidable amount of water originating from the electrolyte solution preparation.

Surprisingly, we observed a completely different behavior when Br^−^ is present in the solution. The hydrogen evolution reaction becomes more prominent for both Au and Ag electrodes in the presence of Br^−^. This observation is consistent with findings by Garg et al., who observed higher HER activity when Br^−^ anion was used in an aqueous solution over an Ag electrode.^[29]^ This increased HER activity results in higher current densities and exhibits faster kinetics than CO_2_RR. We noticed a minimal shift in onset potential between N_2_‐saturated and CO_2_‐saturated electrolytes, suggesting slight changes in reaction conditions. In contrast to Br^−^, the presence of Cl^−^ results in the highest positive shift in onset potential.

Furthermore, the adsorption of chloride ions on an Ag electrode initiates at a potential less negative than that of the bromide anion.^[49]^ Other studies verify that Cl^−^ not only suppresses HER but also enhances CO_2_ selectivity through surface adsorption on electrodes, promoting increased CO production.[^29^, ^42^, ^43^, ^44^] However, this effect is less pronounced for Au electrodes. Eberhardt et al. demonstrated the gradual adsorption process of chloride on Au in aqueous solution, noting the significant strength of this adsorption.^[50]^

The concentration of halide anions near the cathode surface significantly influences the electrochemical properties and structure of the surface. These ions form direct bonds with the electrode surface, impacting the double layer capacitance. Chen et al. demonstrated the beneficial role of Cl^−^ in CO_2_RR by facilitating electron transfer processes. They illustrated that solvated Cl^−^ ions adsorb specifically on the surface, forming bonds with the catalyst electrode. Subsequently, electrons flow from the electrode to the adsorbed Cl^−^, facilitating the reduction of CO_2_ near the electrode surface and at the same time suppressing the hydrogen evolution reaction.[^42^, ^44^]

To further investigate the interactions between different anions and the electrode surface, we conducted qualitative and quantitative surface characterization techniques, including SEM‐EDX and AFM. Our aim was to explore how the electrode surface undergoes restructuring and changes in roughness following CO_2_RR.

**Figure 5:**
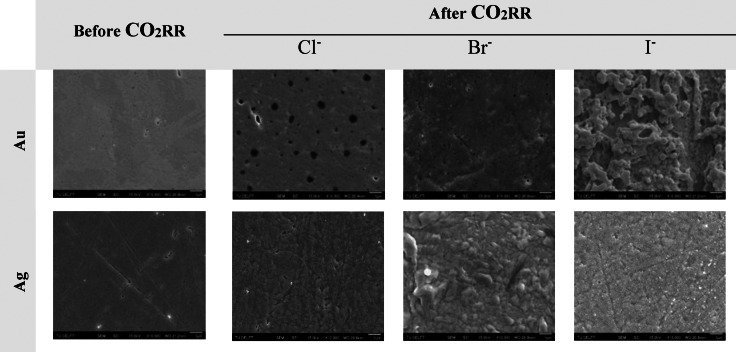
SEM images of electrodes before and after CO_2_RR for 1 hour at −1.5 V vs. Ag/AgCl over Au and Ag electrodes in ChX : EG 1 : 4 (X : Cl^−^, Br^−^, I^−^) electrolytes, scale bar 1 μm.

**Figure 6:**
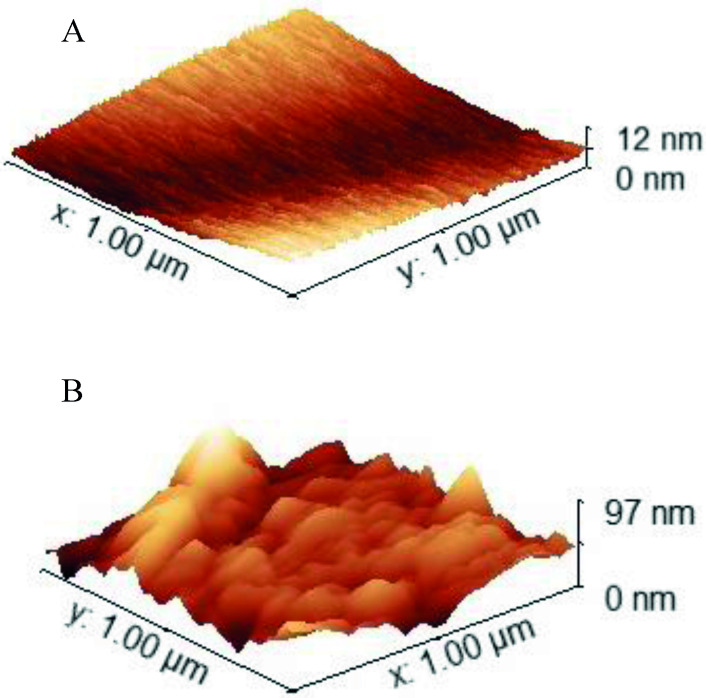
AFM topography images of the Ag surface A) before CO_2_RR, B) after CO_2_RR for 1 hour at −1.5 V vs. Ag/AgCl in ChCl : EG 1 : 4 electrolyte.

In Figure 5, SEM images reveal that both Au and Ag electrodes exhibited rougher surfaces compared to freshly polished ones. Particularly noteworthy is the increased roughness observed on the Ag electrode after CO_2_RR, as evident in comparisons with the Au electrode (Table S7 and S8). This roughened texture is also clearly depicted in the AFM topography images (Figure 6 and S7). The average roughness (R_a_) significantly increased from 1.8 nm for a freshly polished Ag electrode before CO_2_RR to 8.8 nm after CO_2_RR in ChCl (Table S6).

We hypothesize that this roughening of the flat Ag surface is a result of surface reconstruction, driven by the dissolution of Ag oxide layers and their subsequent redeposition on the electrode surface, as noted by Garg et al.^[18]^ Furthermore, we conducted ICP‐OES analysis on the catholyte solutions before and after CO_2_RR experiments, which supported our hypothesis by detecting the dissolution of Ag only after CO_2_RR (Table S5).

Consistent with the electrochemical reduction of CO_2_ on the Ag electrode in ChCl, AFM topography analysis revealed surface reconstruction during CO_2_RR. This reconstructed surface, characterized by increased roughness, facilitates a more distributed surface area and alters the electrochemically active surface area for CO_2_ reduction. Consequently, this enhanced roughness indicates a surface reconstruction process driven by dissolution‐redeposition phenomena at the electrode‐electrolyte interface during CO_2_RR.

### 3.2 Effect of Increasing Organic Solvent Content

Here, we further explore the effect of ethylene glycol content in Cl^−^ containing electrolytes on enhancing current density during CO_2_RR and CO selectivity on the Ag electrode. The ChCl and Ag electrode combination, which exhibited superior CO selectivity in previous experiments, was chosen for this investigation.

**Figure 7:**
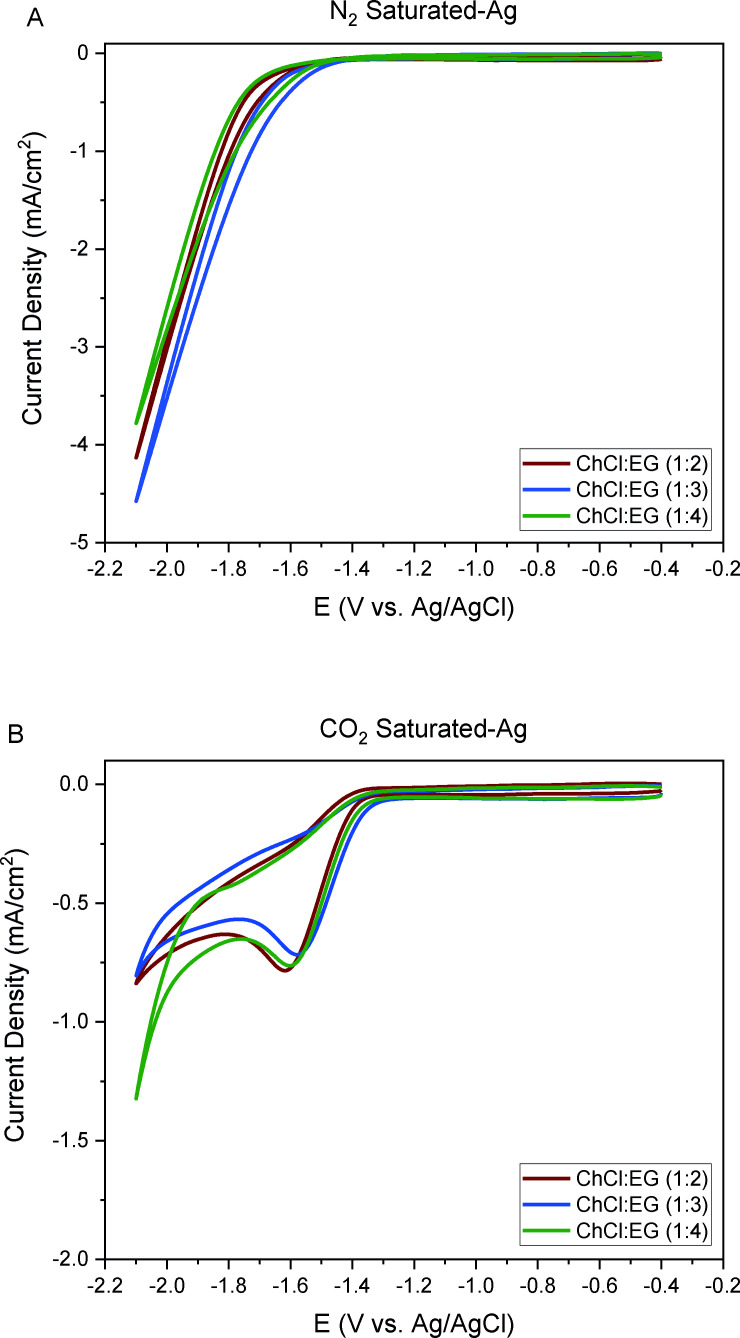
Cyclic voltammograms of ChCl : EG 1 : X (X : 2, 3, 4) electrolytes over Ag electrode in electrolytes saturated with A)  N_2_ , B) CO_2_.

Initially, the performance of the electrolyte at different mole ratios of ethylene glycol and ChCl on Ag was assessed through cyclic voltammetry under electrolytes saturated with N_2_ and CO_2_ (Figure 7). Consistent with previous cyclic voltammograms, a reduction peak was only observed when the electrolyte was saturated with CO_2_.

Upon increasing the mole ratio of EG to ChCl from 2 to 4, no significant shift was observed for CO_2_‐saturated electrolytes (−1.6, −1.55, and −1.6 V vs. Ag/AgCl for ChCl : EG 1 : 2, 1 : 3, and 1 : 4, respectively). However, conducting CPE experiments at −1.5 V vs. Ag/AgCl revealed a slight improvement in total current density with increasing EG content (Table 2). This resulted in a higher partial current density to H_2_ with a relatively similar partial current density of CO (Table S4).

**Table 2:** Electrochemical and physical properties of ChCl : EG 1 : X (X=2, 3, 4) electrolytes. FE was measured at an applied potential of −1.5 V vs. Ag/AgCl over the Ag electrode.

Electrolyte	FE_CO_/FE_H2_	Total current density (mA.cm^−2^)	Viscosity* (mPa.s)	Conductivity* (mS.cm^−1^)
ChCl : EG 1 : 2	17.5	−0.45	29	8.7
ChCl : EG 1 : 3	16.7	−0.51	21	9.6
ChCl : EG 1 : 4	12.2	−0.60	20	9.1

* Physical properties of electrolytes were measured before the controlled potential electrolysis experiments at 25 °C.

The results for ChCl : EG (1 : 2) on an Ag electrode were comparable to those reported by Vasilyev et al., with 78 % FE_CO_ and a total current density of −0.43 mA.cm^−2^.^[37]^ The higher current density in a solution with higher EG content can be attributed to the observed reduction in viscosity and slight improvement in electrical conductivity (Table 2). However, it remained relatively low, likely due to the formation of a thicker hydrodynamic boundary layer at the catalyst surface, limiting efficient mass transport of CO_2_.

Furthermore, increasing the EG mole ratio to 4 resulted in increased CO_2_ solubility due to the increased free volume within the deep eutectic solvent, providing more CO_2_ in the system and leading to enhanced CO formation (FE_CO_=84 %).^[34]^ However, under this condition, HER also competes with CO_2_ reduction, as evidenced by a relatively higher partial current density for H_2_. Additionally, through testing with HPLC, we observed the diffusion of EG to the anode compartment during the reaction (Figure S2). This diffusion resulted in the crossover of water to the cathode compartment, thereby creating an environment conducive to enhanced H_2_ formation.

## 4 Conclusion and Recommendations

In this study, we investigated the electrochemical reduction of CO_2_ using an electrolyte containing choline cation paired with various halide anions (Cl^−^, Br^−^, and I^−^) in ethylene glycol on Ag and Au cathodes. Our findings revealed that the Ag electrode outperformed the Au electrode in terms of CO selectivity. Anion variations demonstrated a notable impact on the electrocatalytic performance in CO_2_RR, with the highest faradaic efficiency for CO observed with Cl^−^ as the counter ion in the electrolytes for both electrodes.

This study led us to conclude that the intrinsic characteristics of Cl^−^ not only suppressed HER but also enhanced the selectivity of CO_2_ through surface adsorption on both Ag and Au electrodes, thereby promoting CO production. On the Ag electrode, Cl^−^ played a significant role in forming an active surface layer, leading to significantly higher partial current density of CO compared to H_2_. This restructuring of surface morphology contributed to the catalytic activity and selectivity of the silver surface, which remained stable after 1 hour with a current density of 0.6 mA.cm^−2^.

To further improve the low current density observed, we explored using different ratios of ethylene glycol in the chloride containing electrolyte over the Ag electrode. While all three ratios showed stable total current densities throughout controlled potential electrolysis experiments, they remained relatively low. Despite the improved viscosity with increased EG content, a thicker hydrodynamic boundary layer at the catalyst surface hindered efficient mass transport of CO_2_. Nevertheless, we speculate that employing a gas‐diffusion electrode (GDE) in a continuous flow‐cell setup and incorporating co‐solvents could further enhance electrolyte performance by decreasing viscosity and increasing CO_2_ solubility, which is currently underway.

This work contributes to provide valuable insights into anion‐induced surface restructuring of Ag and Au electrodes during electrochemical CO_2_ reduction, offering a pathway to optimize electrolyte performance in non‐aqueous solutions.

## Supplementary Data

Supplementary data associated with this article can be found in the supporting information file.

## Conflict of Interests

The authors declare no conflict of interest.

5

## Supporting information

As a service to our authors and readers, this journal provides supporting information supplied by the authors. Such materials are peer reviewed and may be re‐organized for online delivery, but are not copy‐edited or typeset. Technical support issues arising from supporting information (other than missing files) should be addressed to the authors.

Supporting Information

## Data Availability

The data that support the findings of this study are available from the corresponding author upon reasonable request.
