# Supplementary material for: Effect of Halide Anions on Electrochemical CO2 Reduction in Non‐Aqueous Choline Solutions using Ag and Au Electrodes
Source: ChemistryOpen. 2024 Sep 10;13(11):e202400166. doi: 10.1002/open.202400166 (PMC11564868; doi:10.1002/open.202400166)
Supplement: Supplementary file 1 — Supporting Information [file OPEN-13-e202400166-s001.pdf]

ChemistryOpen

Supporting Information

Effect of Halide Anions on Electrochemical CO_2 Reduction in Non-Aqueous Choline Solutions using Ag and Au Electrodes

Hengameh Farahmandazad,* Simone Asperti, Ruud Kortlever, Earl Goetheer, and Wiebren de Jong

Supporting Information for:

Effect of Halide Anions on the Electrochemical CO_2 Reduction in Non-aqueous Choline Solutions using Ag and Au Electrodes

Hengameh Farahmandazad[+], Simone Asperti, Ruud Kortlever, Earl Goetheer, Wiebren de Jong

Section of Large Scale Energy Storage, Process & Energy Department, Faculty of Mechanical Engineering

Delft University of Technology

Address: Leeghwaterstraat 39, 2628 CB, Delft, The Netherlands

E-mail: h.farahmandazad@tudelft.nl

Table of Contents

Water content measurement 3

HPLC analysis 3

Chronoamperometry recorded on Au and Ag electrodes 5

Total current density versus applied potential 5

Faradaic efficiency for H_2 and CO over Au and Ag electrodes 6

Faradaic efficiency versus time for H_2 and CO over Au and Ag electrodes 8

ICP-OES analysis of catholyte and anolyte solutions 9

AFM analysis before and after CO_2RR on Ag and Au electrode 11

SEM-EDS analysis before and after CO_2RR on Au electrode 12

SEM-EDS analysis before and after CO_2RR on Ag electrode 15

Water content measurement

Table S1 shows an example of a typical water content measurement performed using a volumetric Karl-Fischer titrator. The measurement was performed for the catholytes before and after CPE experiments of CO_2RR.

Table S1. Water content measurements by Karl-Fischer titration of catholyte before and after the CPE experiments of CO_2RR.

Catholyte sample	Water content (Wt%)	STD (%)
Pre CO_2RR, ChCl:EG (1:2)	0.26	0.04
Post CO_2RR, ChCl:EG (1:2), -1.4V vs. Ag/AgCl	5.87	0.88
Post CO_2RR, ChCl:EG (1:2), -1.5V vs. Ag/AgCl	5.51	0.48
Post CO_2RR, ChCl:EG (1:2), -1.6V vs. Ag/AgCl	5.76	0.39

HPLC analysis

HPLC analysis was performed on a catholyte and anolyte solution before and after CO_2RR. In Figure S1 and Figure S2, a typical HPLC spectrum with corresponding retention time for choline, formate, ethylene glycol, and sulfuric acid is shown. The HPLC was calibrated with standard aqueous solutions in a range of 0.1 mM to 50 mM for liquid products, including oxalic acid, glyoxal, formate, acetic acid, ethylene glycol, acetaldehyde, methanol, ethanol, acetone, propionaldehyde, 2-propanol, 1-propanol, choline chloride, and acetic acid. None of these liquids was detected as products after CO_2RR. This analysis confirmed that a crossover of ethylene glycol toward the anolyte compartment via the membrane was taking place.

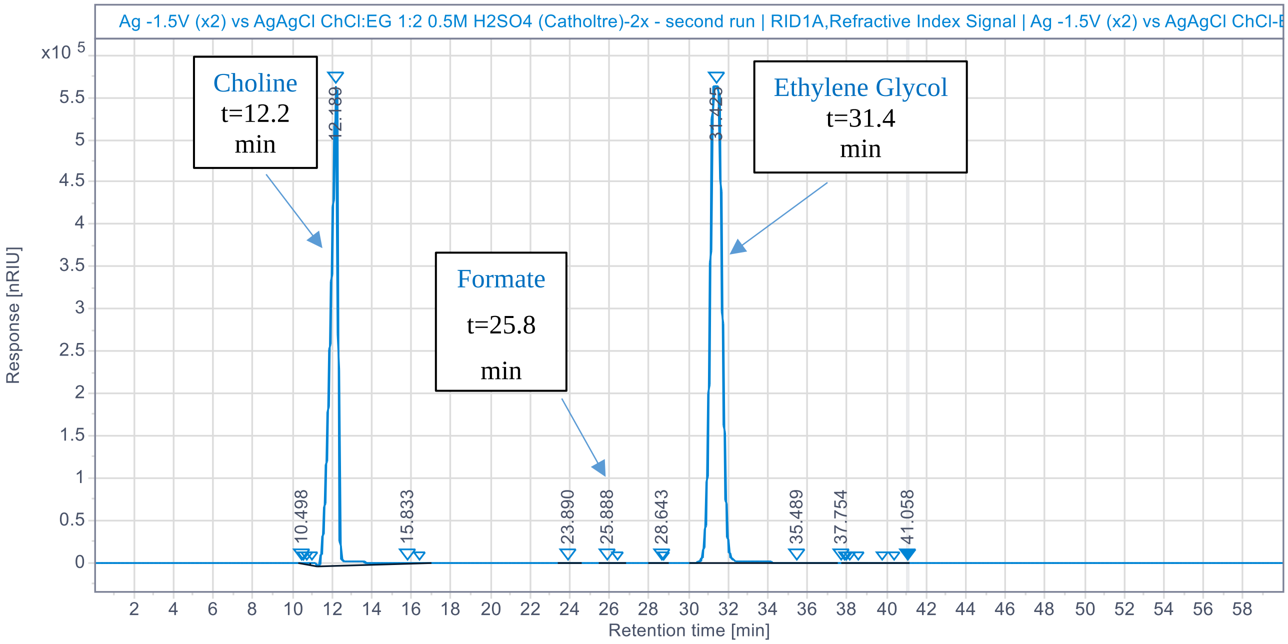

Figure S1. HPLC spectrum of catholyte, ChCl:EG (1:2), Post CO_2RR at -1.5V vs. Ag/AgCl on Ag electrode.

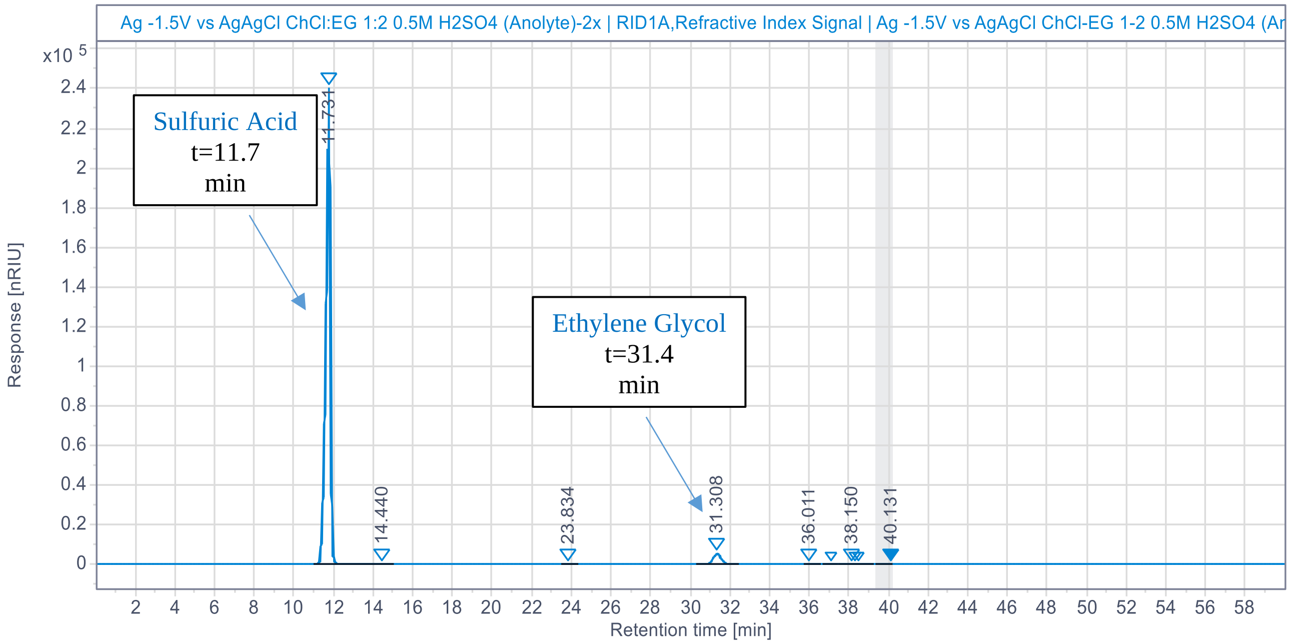

Figure S2. HPLC spectrum of anolyte, 0.5M H_2SO_4, Post CO_2RR at -1.5V vs. Ag/AgCl on Ag electrode.

Chronoamperometry recorded on Au and Ag electrodes

Examples of a typical chronoamperometry experiments recorded during CO_2RR over Au and Ag electrodes in ChX:EG 1:4 (X:Cl^-, Br^-, I^-) catholytes at -1.5 V vs. Ag/AgCl are shown in Figure S3. Clearly, the currents recorded were more negative at higher applied potentials, where more redox processes take place, i.e., more electrons are involved during the process and lead to an increase in the total current.

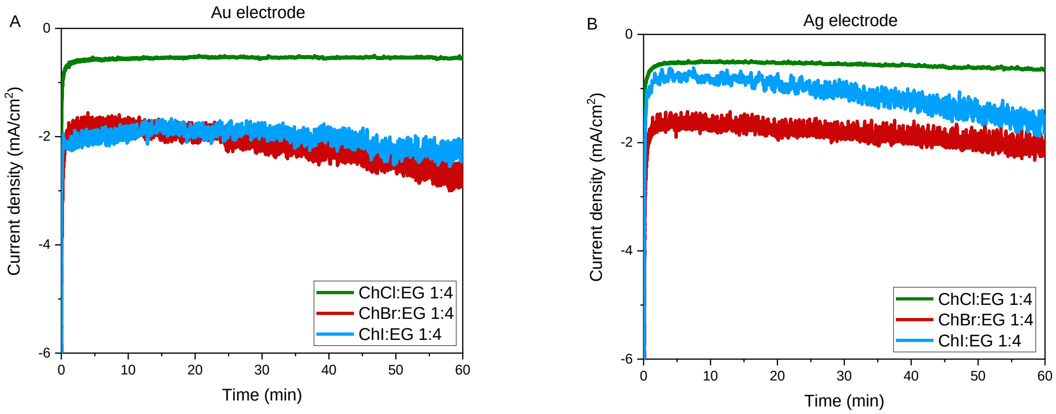

Figure S3. Current density vs. time data during chronoamperometry experiment for 1 hour at -1.5 V vs. Ag/AgCl in ChX:EG 1:4 (X:Cl^-, Br^-, I^-) over A) Au electrode and B) Ag electrode.

Total current density versus applied potential

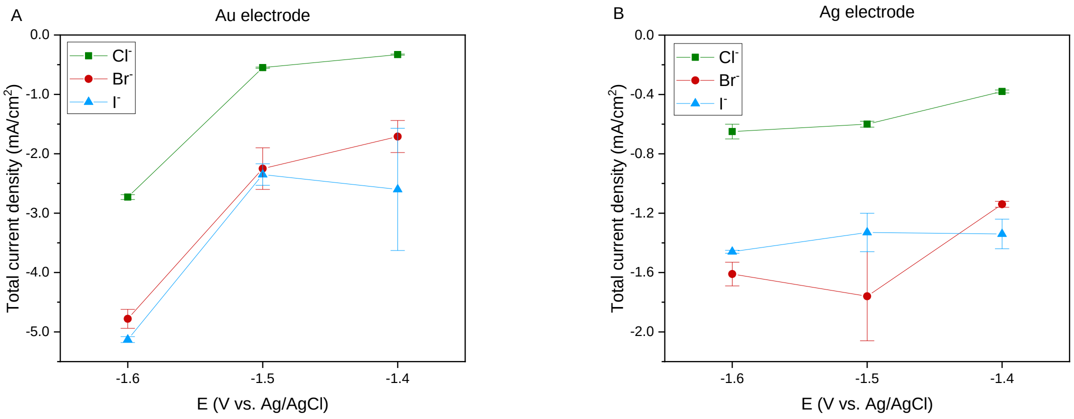

Figure S4. Total current density vs. potential: A) Au electrode B) Ag electrode in ChX:EG 1:4 (X:Cl^-, Br^-, I^-) catholytes after 1 hour of CPE experiments.

Faradaic efficiency for H_2 and CO over Au and Ag electrodes

Table S2. Average of FE_{H2} and FE_{CO} over Au electrode after 1 hour of CPE experiments.

Potential (V vs. Ag/AgCl)	Anion type	FE_{H2} (%)	STD_{H2} (%)	FE_{CO} (%)	STD_{CO} (%)
-1.4	Cl^-	54.5	4	30.8	6.9
-1.5		35.1	5.5	61.6	1.3
-1.6		94.3	3.2	0.9	0.6
-1.4	Br^-	91.9	2.9	0	0
-1.5		96.6	2.3	0	0
-1.6		95.0	0.7	0	0
-1.4	I^-	79.9	5.6	9.4	3.8
-1.5		77.9	0.6	15.2	2.2
-1.6		94.8	1.7	0.6	0.7

Table S3. Average of FE_{H2} and FE_{CO} over Ag electrode after 1 hour of CPE experiments.

Potential (V vs. Ag/AgCl)	Anion type	FE_{H2} (%)	STD_{H2} (%)	FE_{CO} (%)	STD_{CO} (%)
-1.4	Cl^-	10.2	2.3	79	3.3
-1.5		6.9	1.9	83.7	0.3
-1.6		11.5	4.0	82.0	0.7
-1.4	Br^-	90.7	0.5	0.5	0.5
-1.5		91.9	4.8	0.2	0.3
-1.6		92.9	2.5	0.1	0.1
-1.4	I^-	88.3	1.4	4.9	0.7
-1.5		46.4	2.2	47.3	0.3
-1.6		21.4	5.2	71.7	7.6

Table S4. Average of FE_{H2} and FE_{CO} and partial current density over Ag electrode with different EG content; ChCl:EG 1:X (X = 2, 3, 4), after 1 hour of CPE experiments at -1.5V vs. Ag/AgCl.

Electrolyte	FE_{H2} (%)	STD_{H2} (%)	FE_{CO} (%)	STD_{CO} (%)	j_{H2} (mA.cm^{-2})	STD_{jH2} (mA.cm^{-2})	j_{CO} (mA.cm^{-2})	STD_{jCO} (mA.cm^{-2})
ChCl:EG 1:2	4.7	0.3	82.4	4.4	-0.02	0.00	-0.38	0.03
ChCl:EG 1:3	4.5	1.6	75.2	3.5	-0.03	0.01	-0.42	0.02
ChCl:EG 1:4	6.9	2.0	83.7	0.3	-0.04	0.01	-0.50	0.02

Faradaic efficiency versus time for H_2 and CO over Au and Ag electrodes

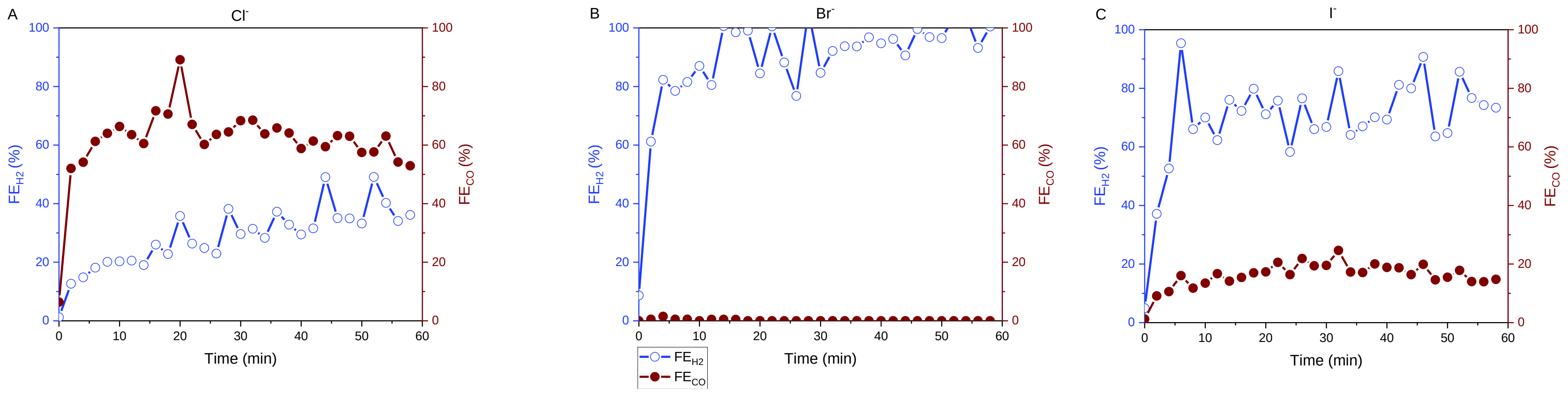

Figure S5. Time-dependence of the faradaic efficiency of H_2 and CO detected in ChX:EG 1:4 X: A)Cl^-, B)Br^-, and C) I^-electrolytes during 1 hour of CPE experiments at -1.5 V vs. Ag/AgCl over Au electrode.

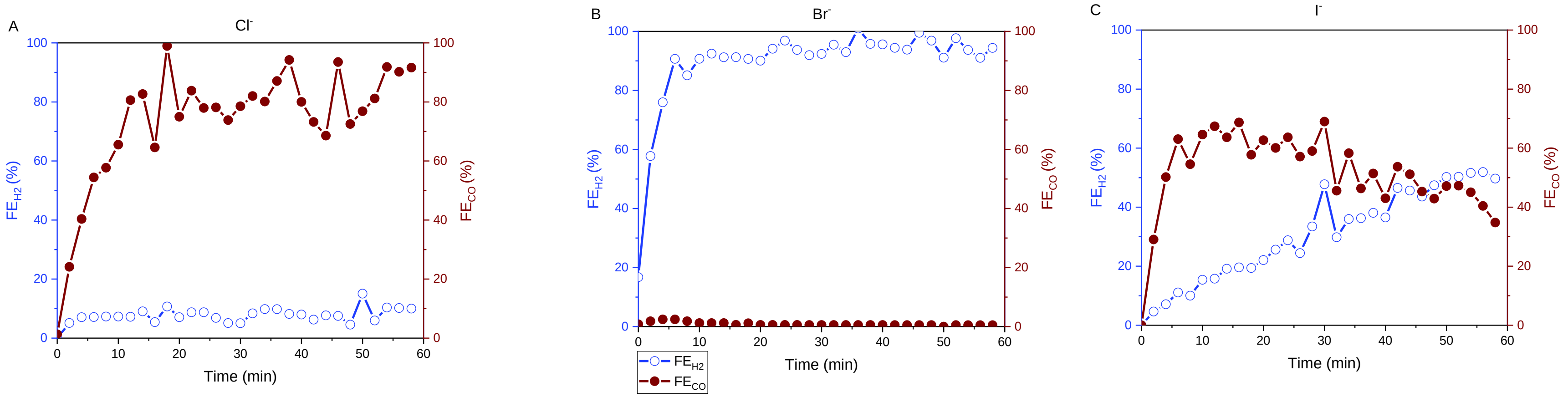

Figure S6. Time-dependence of the faradaic efficiency of H_2 and CO detected in ChX:EG 1:4 X: A)Cl^-, B)Br^-, and C) I^-electrolytes during 1 hour of CPE experiments at -1.5 V vs. Ag/AgCl over Ag electrode.

ICP-OES analysis of catholyte and anolyte solutions

ICP-OES tests were performed to find out the possible sources of elements which are present in the system. Some of these elements were also detected on the electrode surface via the EDS measurements, including Al, Si, and K (See Table 6-7). Al and Si elements were detected in the choline-solution and ethylene glycol itself by ICP-OES as impurities. Also for the samples from the Ag electrodes, these two elements come also from the polishing treatment using alumina paste and silicon paper. We saw that these two elements are present in the catholyte after CO_2RR. This confirms the crossover from the catholyte to the anolyte compartment during CO_2RR of these two elements. Probably, the presence of Al in the anode compartment lead to aluminum oxide, which could have ended up on deposition on the membrane and anode, negatively affecting the efficiency of the system. As it can be seen in Table S5 no Cl and Br elements were detected in the samples corresponding to Br-containing and Cl-containing solutions, respectively.

Table S5. ICP-OES measurements of EG, ChCl, ChBr. catholyte and anolyte before and after the controlled potential experiments of CO_2RR. All values are in mg/kg.

Sample name	Ag	Al	Ca	Cu	Fe	K	Mg	Mn	Na	Zn	Si	Cl	Br	SO4
EG_blank	-	0.06	0.18	-	-	-	-	-	-	-	1.76	-	-	-
ChBr_blank	-	0.24	2.13	0.24	0.24	3.32	2.37	0.24	38.15	3.79	7.58	-	236937.13	-
ChBr:EG_Catholyte_PreCO_2RR	-	0.00	0.82	0.14	0.14	0.75	1.02	-	4.21	1.49	0.75	-	67937.26	-
ChBr:EG-H_2SO_4_Anolyte_PostCO_2RR_Ag	-	0.51	1.88	-	0.09	4.54	0.09	-	0.17	-	3.68	-	-	85643.72
ChBr:EG-H_2SO_4_Catholyte_PostCO_2RR_Ag	23.44	0.07	2.00	0.40	0.13	4.21	1.00	-	8.95	1.54	1.47	-	66789.26	-
ChCl_blank	-	0.17	11.60	-	1.02	0.85	3.07	-	30.03	0.34	8.70	170651.20	-	-

ChCl:EG_Catholyte_PreCO_2RR	-	0.06	1.87	-	0.44	-	1.12	-	1.37	-	0.62	62177.49	-	-
ChCl:EG-H_2SO_4_Anolyte_PostCO_2RR_Ag	-	0.56	2.80	0.24	0.08	0.56	0.08	-	-	0.08	1.92	-	-	80016.53
ChCl:EG-H_2SO_4_Catholyte_PostCO_2RR_Ag	28.28	0.06	3.40	0.35	0.40	0.35	1.10	-	2.08	0.17	0.63	57709.61	-	-

\- indicates that the data were not detected

AFM analysis before and after CO_2RR on Ag and Au electrode

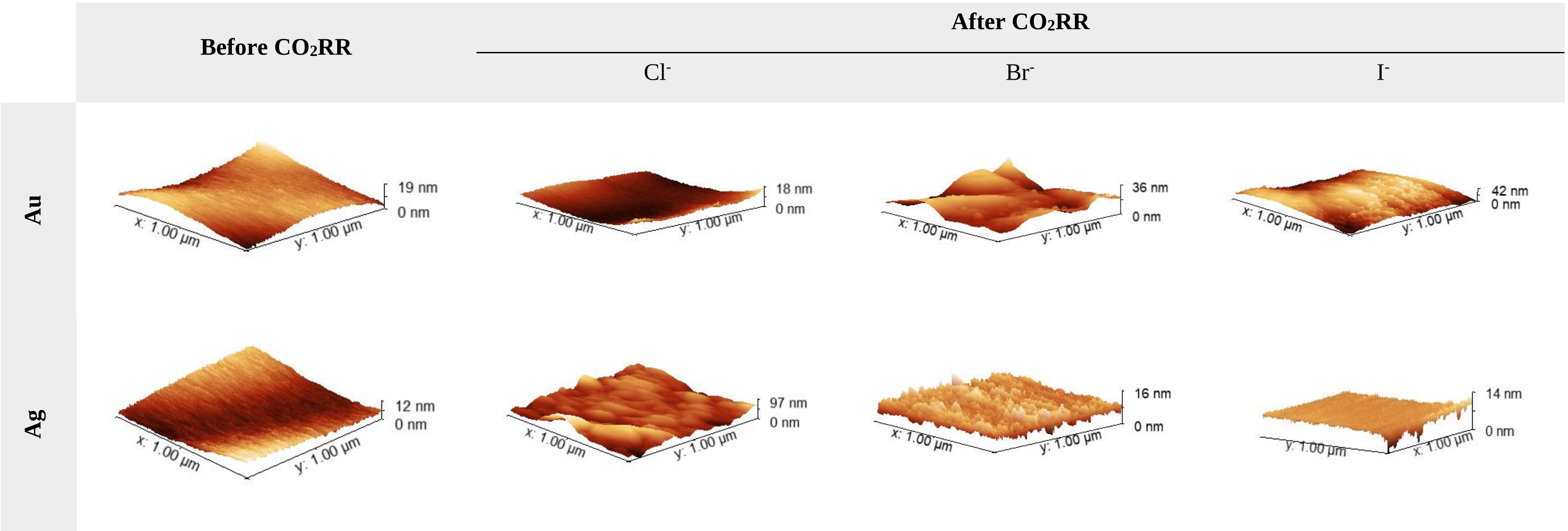

Figure S7. AFM topography images of electrodes before and after CO_2RR for 1 hour at -1.5V vs. Ag/AgCl over Au and Ag electrodes in ChX:EG 1:4 (X:Cl^-, Br^-, I^-) electrolytes.

Table S6. Average roughness (R_a) of a surface measured by AFM.

**Electrode type**	**R_a before CO_2RR (nm)**	**R_a after CO_2RR** (nm)		
		Cl^-	Br^-	I^-
**Au**	1.8	1.8	3.9	6.0
**Ag**	1.8	8.8	1.1	0.3

SEM-EDS analysis before and after CO_2RR on Au electrode

To test the morphology changes during CO_2RR, additional SEM-EDS tests were performed on Au electrode foil with the below specification. EDS was employed after recording SEM images.

Data Type:	Counts
Image Resolution:	1024 by 812
Image Pixel Size:	0.01 µm
Map Resolution:	256 by 202
Map Pixel Size:	0.06 µm
Acc. Voltage:	15.0 kV
Magnification:	8000
Detector:	Ultradry

Results reported in Table S7, indicated high purity and cleanliness of the Au surface electrode in pre-CO_2RR.EDS elemental analysis showed also the efficacy of the treatment procedure employed in this work, since no major impurities were present. The presence of carbon could be associated to a leftover due to the flame annealing treatment involved. After CO_2RR in all ChX:EG 1:4 (X: Cl^-, Br^-, I^-) solutions, no other elements were detected on the surface.

Table S7. EDS analysis on Au electrode Pre-CO_2RR and Post-CO_2RR for in ChX:EG 1:4 (X: Cl^-, Br^-, I^-) at -1.5V vs. Ag/AgCl.

Condition	EDS elemental mapping

Pre-CO_2RR	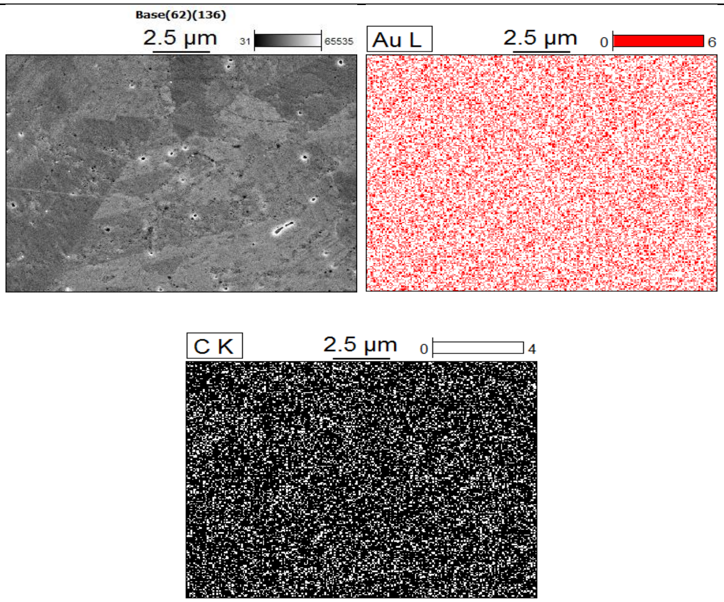	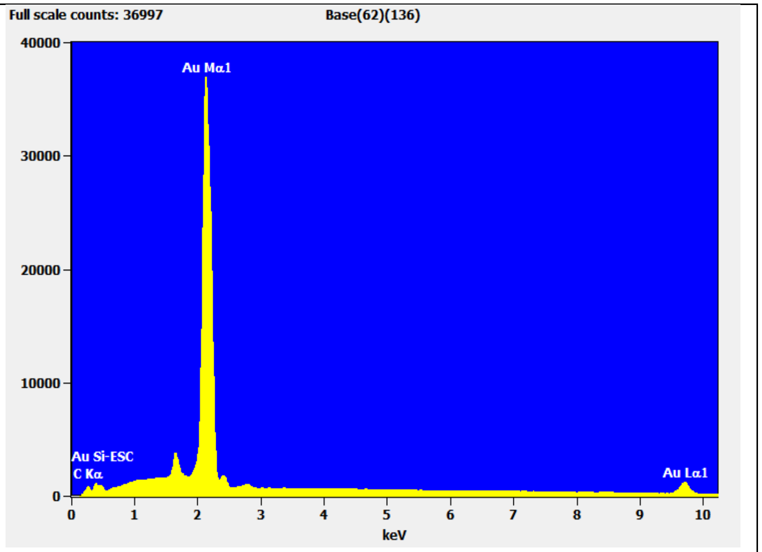
Post-CO_2RR<br>X:Cl^-	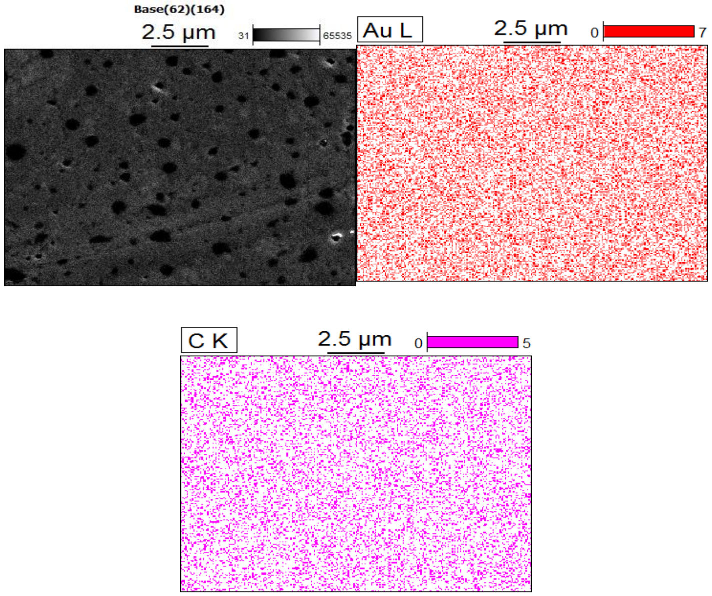	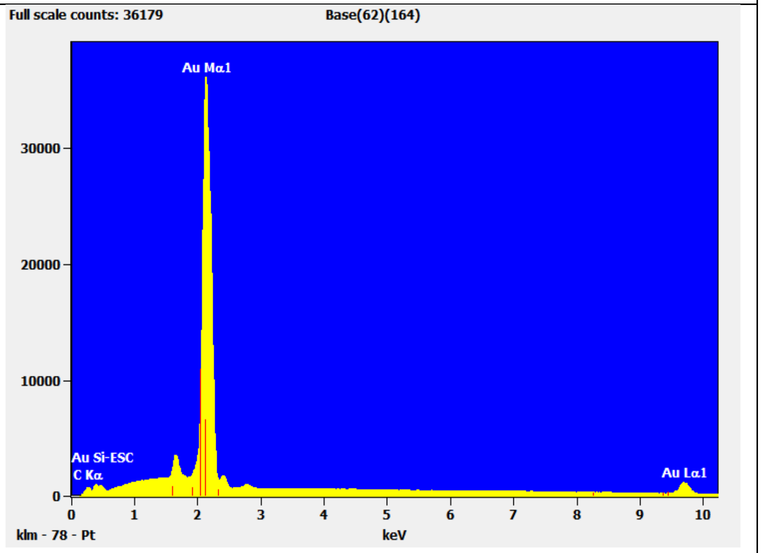

Post-CO_2RR<br>X:Br^-	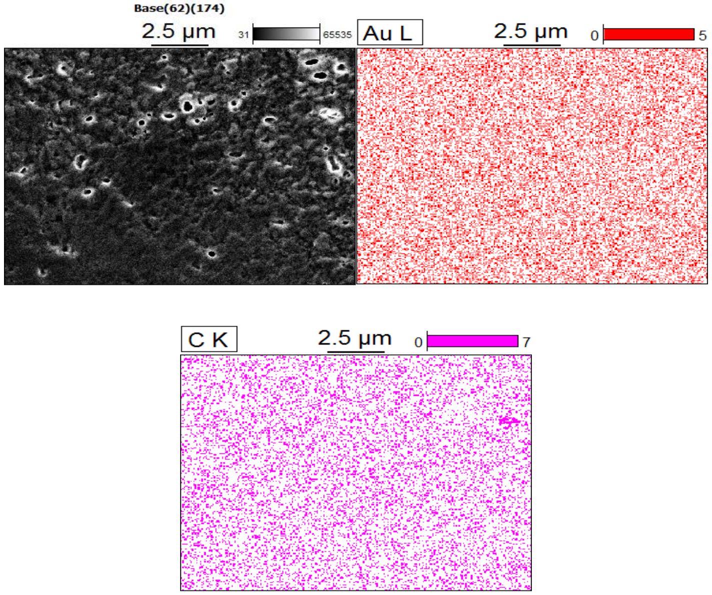	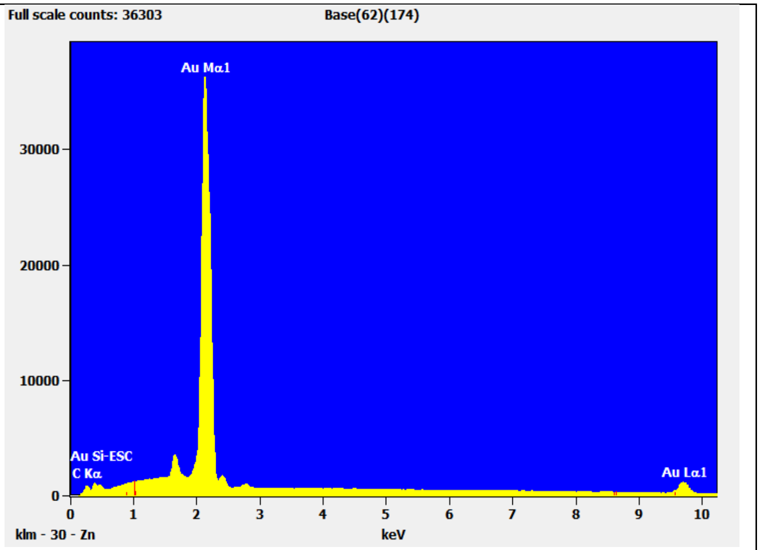
Post-CO_2RR<br>X:I^-	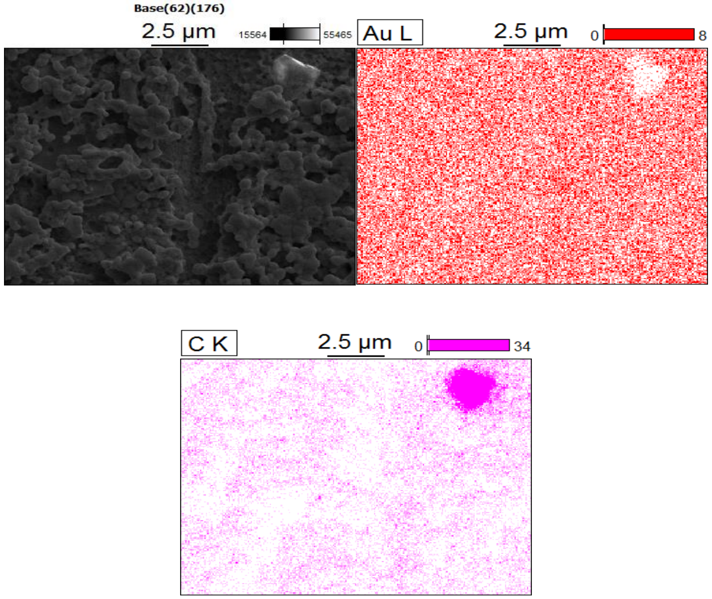	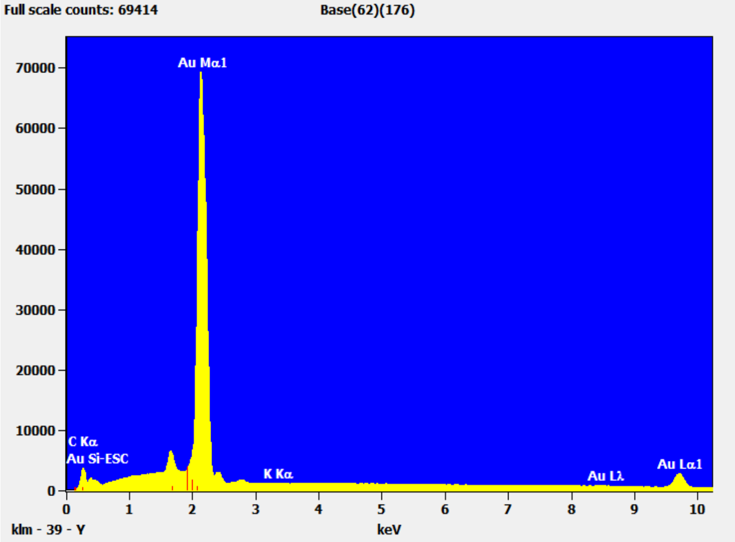

SEM-EDS analysis before and after CO_2RR on Ag electrode

Similarly to the tests performed on Au foils, additional SEM-EDS tests were performed on Ag foil with the same experimental settings. According to Table S8, EDS analysis performed on the Ag surface electrode in pre-CO_2RR and post-CO_2RR conditions detected mainly Ag, K, Si, and Cl as sure elements. The signals for Si, K, and Cl had low intensity and, therefore, we considered the presence of such elements on the catalyst surface negligible. A confirmation test was performed using XPS analysis, which showed in Figure S8. We believe that Cl derives from the manufacturing process and, probably, it is not related to the electrochemical processes. Instead, the traces of Si could be linked to the polishing sandpaper step, which was used in the polishing treatment. Similar to the Au electrode, no other elements were detected on the surface of the Ag electrode after CO_2RR in all ChX:EG 1:4 (X:Cl^-, Br^-, I^-) solutions, which means, once again, the high purity and cleanliness of the catalyst surface during CO_2RR

Table S8. EDS analysis on Ag electrode Pre-CO_2RR and Post-CO_2RR for in ChX:EG 1:4 (X: Cl^-, Br^-, I^-) at -1.5V vs. Ag/AgCl.

<table>
<tr><th>Condition</th><th colspan="2">EDS elemental mapping</th></tr>
<tr><td>Pre-CO_2RR</td><td>
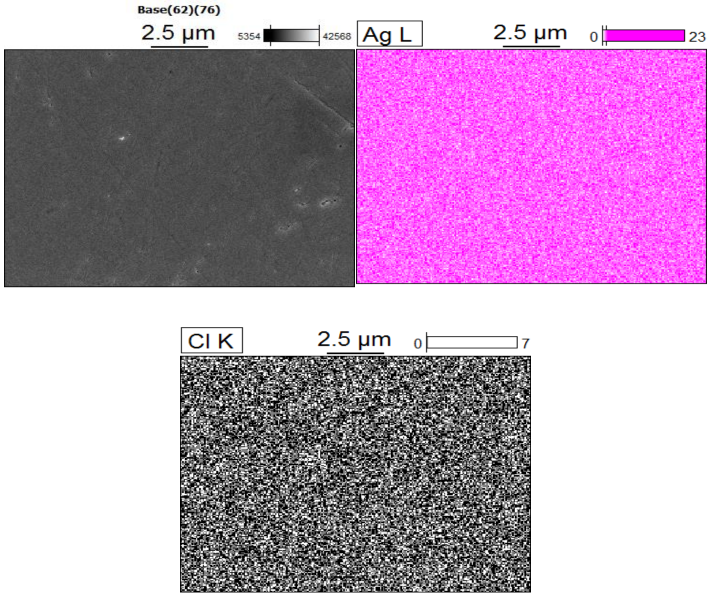

</td><td>
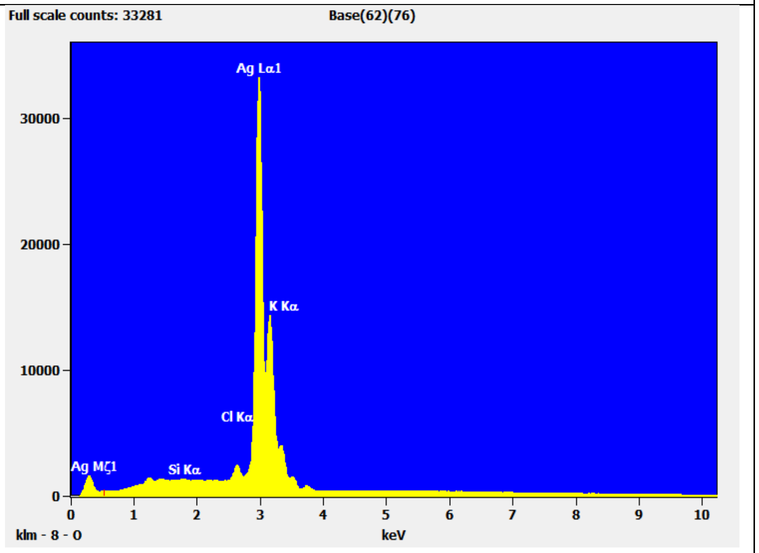

</td></tr>
</table>

<table>
<tr>
<td>Post-CO_2RR

X:Cl^-</td>
<td>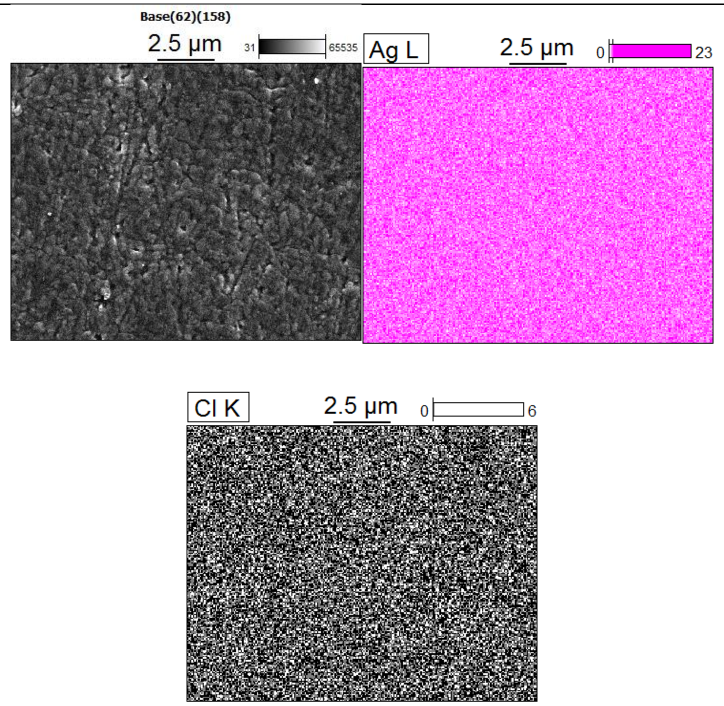
</td>
<td>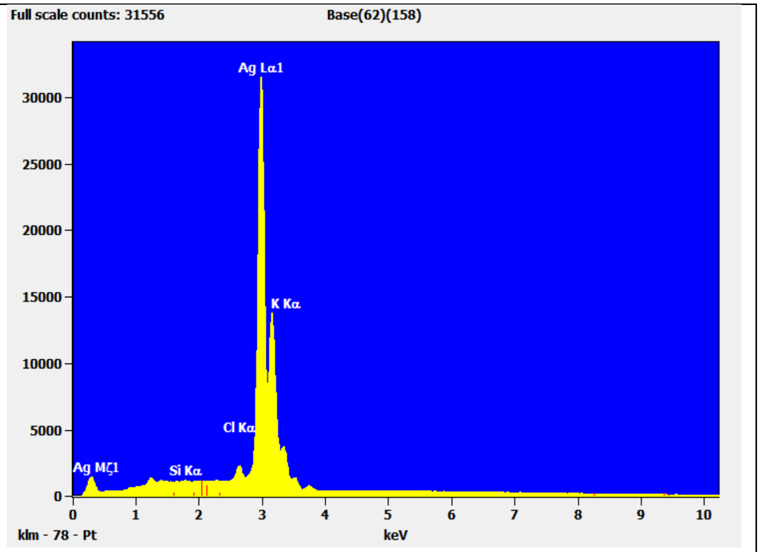
</td>
</tr>
</table>

Post-CO_2RR<br>X:Br^-	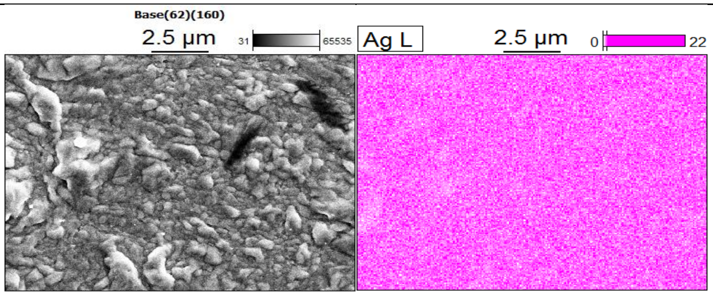	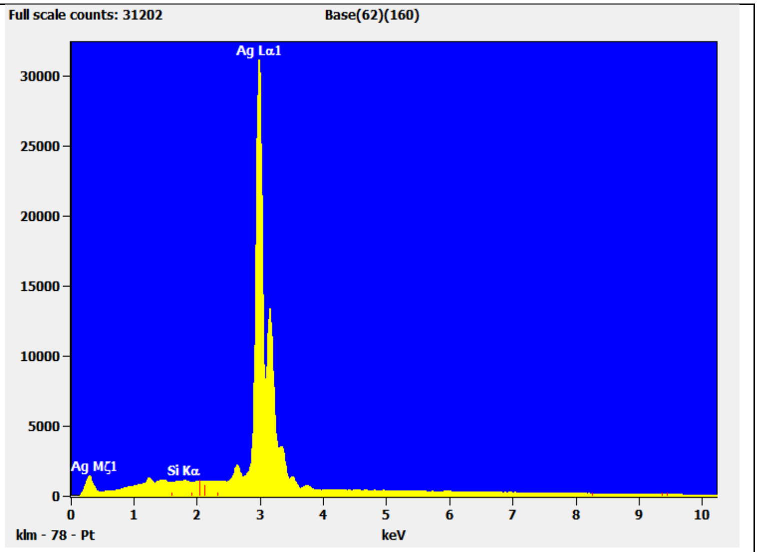
Post-CO_2RR<br>X:I^-	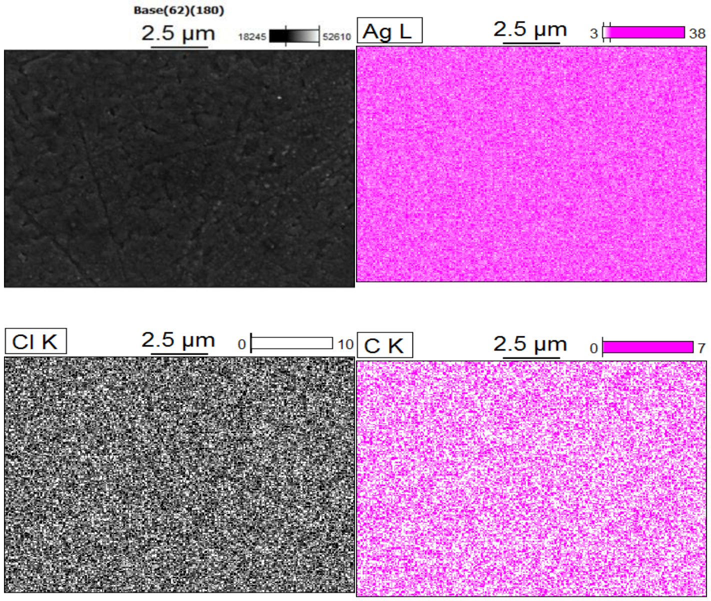	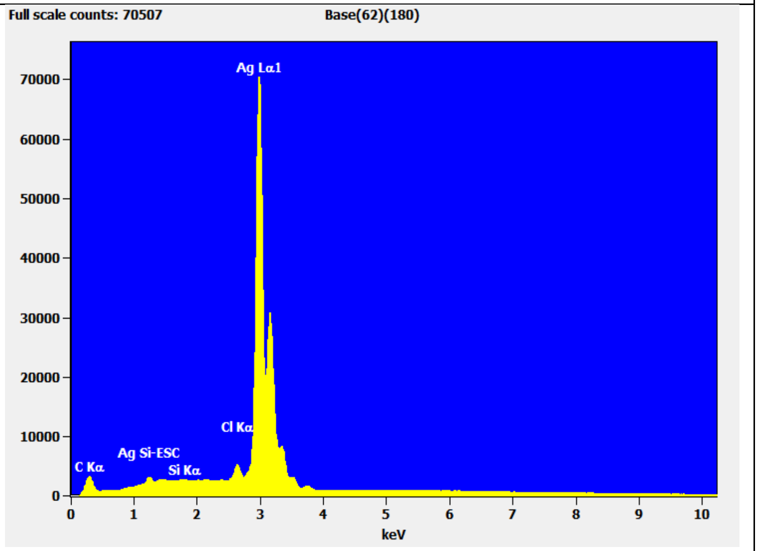

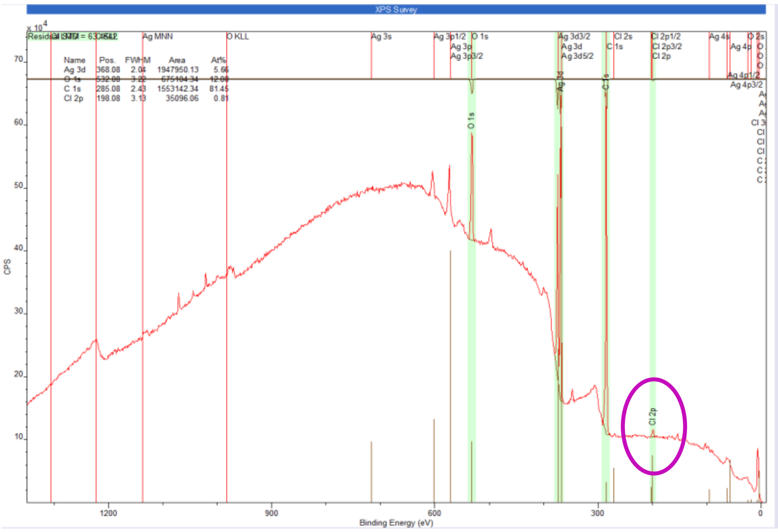

Figure S8. XPS analysis on new-branded Ag electrode before CO_2RR.
